# Transitions in mental health care: The European Psychiatric Association contribution to reform in Bulgaria

**DOI:** 10.1192/j.eurpsy.2020.43

**Published:** 2020-05-05

**Authors:** Julian Beezhold, Drozdstoy Stoyanov, Vladimir Nakov, Helen Killaspy, Wolfgang Gaebel, Zahari Zarkov, Hristo Hinkov, Silvana Galderisi

**Affiliations:** 1 Norwich Medical School, University of East Anglia, Norwich, United Kingdom; 2 Department of Psychiatry and Medical Psychology, Neuropsychiatry and Brain Imaging Group, Medical University of Plovdiv, Plovdiv, Bulgaria; 3 Department of Mental Health, National Center of Public Health and Analyses (NCPHA), Sofia, Bulgaria; 4 Department of Rehabilitation Psychiatry, UCL Division of Psychiatry, London, United Kingdom; 5 Department of Psychiatry and Psychotherapy, WHO Collaborating Center, LVR-Klinkum Düsseldorf, Heinrich-Heine-University Düsseldorf, Düsseldorf, Germany; 6 Department of Mental Health, National Center of Public Health and Analyses (NCPHA), Sofia, Bulgaria; 7 Department of Mental Health, National Center of Public Health and Analyses (NCPHA), Sofia, Bulgaria; 8 Department of Psychiatry, University of Campania “Luigi Vanvitelli”, Naples, Italy

**Keywords:** Bulgaria, European Psychiatric Association, mental health services, quality improvement

## Abstract

**Background:**

The Bulgarian Ministry of Health invited the European Psychiatric Association (EPA) to evaluate Bulgarian mental health care service provision in 2018. Bulgarian mental health services face very significant challenges including a legacy of historic underfunding, internal conflicts, poor planning, and the emigration of very high numbers of younger skilled staff that had followed accession to the European Union. There were significant disputes between stakeholders regarding the way forward and had been at least two unsuccessful previous external agency interventions that had attempted to find solutions.

**Method:**

This EPA position paper describes in detail the EPA mission to Bulgaria including methodology, findings, recommendations, and finally the positive actions and changes that are now underway as a result of the EPA report and intervention aimed at contributing towards improving Bulgarian mental health services.

**Results:**

After meetings with multiple stakeholders in the Bulgarian mental health system and analysis of data on service delivery, workforce, funding and configuration the EPA Panel agreed a list of twenty recommendations for change.

**Conclusions:**

The EPA mission, with the collaboration of multiple stakeholders in Bulgaria, was successful in stimulating high level government action to improve mental health services. Despite longstanding differences, it was possible to involve the stakeholders in constructive dialogue. The importance of “speaking with one voice” was a key lesson learned.

## Aims and Background

In 2018, the European Psychiatric Association (EPA) was invited to send an official Panel, comprising Silvana Galderisi, Wolfgang Gaebel, Helen Killaspy, and Julian Beezhold, to visit and review mental health services in Bulgaria and provide the Ministry of Health with expert recommendations for change needed. The aim of this visit was to provide guidance and impetus to help achieve more consensus and, importantly, to allow much needed reforms in mental health services to be delivered. The final report was presented to the Bulgarian Government and other stakeholders and is published online on the EPA website [1].

Bulgaria has around 7 million inhabitants distributed over 110,879 km^2^ including small Turk and Roma populations, and joined the European Union (EU) in 2007. Bulgaria ranks 75th in the world by per capita GDP. The Health Act 2005 was introduced following the fall of communism, before which health service provision reflected Soviet practice. No Mental Health Care Act has been introduced this far, despite some efforts in that direction.

Mental health services in Bulgaria, by common consensus among the Ministry of Health, the Bulgarian Psychiatric Association, medical, nursing and other staff, patients, and families, are in an unsatisfactory situation and there is a pressing need for reform.

Attempts at reform had stalled due to the complexity of funding arrangements and divergent stakeholder opinions.

There had been two previous WHO missions to advise the government on recommended changes, but these had not led to successful reforms [2,3].

## Process: Information and Documents considered by the EPA Advisory Panel

The EPA Panel consulted widely with many stakeholders, as well as policymakers and politicians, visited a range of Bulgarian mental health services and took note of written information and previous recommendations from other parties.

These consultations included both formal and informal discussions with a wide range of stakeholders in the Bulgarian mental health system including the Deputy Minister of Health, National Advisors to the Ministry, National Center for Public Health and Analyses, World Health Organization, Bulgarian Psychiatric Association, College of Private Psychiatry of Bulgaria, University Professors, Global Initiative for Psychiatry, and psychiatrists, psychiatrists in training, nursing staff, occupational therapists, social workers, support workers, and patients from State Psychiatric Hospitals (SPHs), Mental Health Centers (MHCs), University Psychiatric Clinics, and nongovernmental organizations.

Documents, discussions, and presentations considered included:

1. Mental health legislation in Bulgaria—a brief overview [4].
2. European Psychiatric Association procedure of observation and evaluation system of mental health services in Bulgaria: self-evaluation report.
3. WHO brief assessment of Bulgarian mental health services 2015.
4. WHO brief assessment of the Bulgarian mental health system 2018.
5. Republic of Bulgaria: “National strategy for long-term care 2017.”
6. Republic of Bulgaria: “Action plan for the period 2018–2021 for the implementation of the national strategy for long-term care.”
7. Mental health services in Bulgaria (presentation by Vladimir Nakov) 2018.
8. Data on income, expenditures, activities, and economic indicators in Bulgarian mental health services—Bulgarian National Center for Public Health and Analyses 2018.

Visits to a number of mental health services and centers in the Sofia region were conducted:

1. Mental Health Center “Sofia County.”
2. University Hospital “Älexsandrovska.”
3. National Center for Public Health and Analyses.
4. State Psychiatric Hospital “St. Ivan Rilski.”
5. University Hospital for Neurology and Psychiatry “St. Naum.”
6. Daily Center “Slatina.”
7. Mental Health Center “Prof. N. Shipkovenski.”

Along with:

1. Interviews and conversations with staff and patients during the course of our visits.
2. Meeting with the Deputy Minister for Health Svetlana Yordanova at the Ministry for Health.
3. Interactive workshop with approximately 20 representatives of stakeholders from the organizations listed above.

It is important to note that all types of services established by law in Bulgaria have been visited. There is no representation of all kinds of psychiatric institutions outside of the Sofia region. It is also important to emphasize that about one-third of the Bulgarian population inhabits this region due to heavy emigration and urbanization processes over the past two decades.

## Findings: Summary of the information collected regarding Bulgarian Mental Health Services

Very detailed information was collected and considered under the following headings:

### Brief history of Bulgarian mental health services

Mental health service provision in Bulgaria can be traced back to the foundation in 1084 of a refuge for mentally ill people in the Bachkovo Monastery. The first qualified psychiatrist was Petar Protich who worked in Romania from 1846 [5]. A dedicated psychiatric ward was opened in 1888 by the psychiatrists N. Moskov, B. Chakalov, G. Paiakov, and D. Vladov, leading to the founding of psychiatry and psychiatric care as a medical specialty in Bulgaria. A short while later, the first known female psychiatrist in Bulgaria, Anastasia Golovina established a psychiatric ward in Varna.

### Legal framework

Legislation impacting on mentally ill people dates back to at least 1905 in Bulgaria. However, the main Bulgarian laws now in force date back to the 1960s and 1970s. Legislative discussion was triggered by the EU accession process from 2001 onwards including the need for improved safeguards for detained patients.

The earlier Peoples’ Health Act and the subsequent 2005 Health Act both incorporate provisions relating to mental health. The 2005 Act explicitly states that persons with mental disorders are entitled to treatment and care equal with the conditions for patients with other diseases. Available funding does not allow for full implementation of the letter and spirit of the Act.

The legal provisions regarding compulsory admission and treatment are broadly similar to those in many other European countries. A brief summary of these provisions is provided hereafter.

Individuals with mental disorders or intellectual deficits that cannot care for their own affairs and are incapacitated are provided for under a specific legal procedure. In cases of criminal acts, perpetrators, who offended in a state of insanity due to mental disorder (excluding personality disorders) or due to some form of addiction (Article 89 and Article 92 of the Penal Code), may receive court ordered compulsory treatment in an ordinary psychiatric hospital or a specialized hospital with a forensic psychiatry unit.

In all Penal Code cases after 6 months compulsory admission the court should either order termination, or continuation of the compulsory treatment or its replacement with voluntary treatment.

Mandatory treatment may be imposed by court order in cases of potentially harmful behavior (aggressive or auto-aggressive risk) under the general Health Act.

There is a special chapter in the Health Act concerning patients’ rights, with detailed rules about informed consent; while involuntary treatment of psychiatric patients is regulated in chapter V. Provisions include:

1. Detention for expert hospital assessment is possible only by court decision (but not by administrative prosecutor decision alone).
2. Differentiation between obligatory detention and obligatory treatment is made.
3. There is differentiation between criteria for involuntary treatment and capacity to give informed consent for treatment, with the consequent requirement for two separate expert assessments and a court ruling.
4. Definition of the conditions for treatment when a patient is detained for expert assessment.
5. Definition of the conditions for the application of temporary physical restraint, and for emergency care.
6. Limitation of all procedural deadlines to two court trial meetings: the first is to review the applications and order expert assessment, and the second to review the expert report and decide on mandatory detention and/or treatment, and assignment of a person to provide informed consent on behalf of the patient if his capacity is judged to be disturbed.
7. Preliminary hospitalization without the requirement for expert assessment is allowed for up to 72 h with permission from a judge.
8. According to the Health Act (excluding Penal Code cases—see above), orders for involuntary treatment for people with mental disorder must be re-assessed after a maximum of 3 months.

Important prerequisites for involuntary treatment, as formulated in the most recent Recommendation 10 (2004) of the Council of Europe, are missing:

1. the admission should have a therapeutic purpose;
2. no less restrictive alternative for providing appropriate care is available; and
3. the opinion of the patient has been taken into consideration.

Another problem concerns capacity to give informed consent. Patients who have been detained and treated involuntarily (whose capacity should be assessed according to the law) represent a small portion of all patients admitted to psychiatric hospitals. The majority of hospitalized patients are deemed competent, while there is serious doubt about or evidence of de facto limited capacity for giving consent.

Article 147 (in force since 2009) mandates the Ministry of Health to maintain a register for persons with mental disorders. The register is for use in assessing fitness to carry a weapon or handling of hazardous materials. The Panel was informed that a separate ordinance to detail this requirement and limit any possible abuse and violation of privacy rights of patients is currently under discussion.

### Policy framework

Health reforms in 2000 introduced market elements largely mediated through a health insurance system. In psychiatry, the new conditions benefited mainly those working in outpatient care, where the processes of market service delivery and decentralization were the same as in most other medical disciplines. A cost-effectiveness evaluation of the overall portfolio of inpatient and outpatient psychiatric services has not been carried out. Assessment and interventions are paid per patient, regardless of the complexity of the case and procedures performed.

Unfortunately, psychiatry in hospital inpatient settings remained outside of these processes and thus largely maintained its institutional character. The lack of funding and managerial will to implement the objectives set out in a number of strategic and policy documents appears to have led to distortions and inequality in mental health services. As a result, the principles of continuity of care, comprehensive service provision, and ongoing supportive care have been difficult to deliver.

### Infrastructure

There are approximately 58 psychiatric beds per 100,000 population in Bulgaria—roughly the European average [6].

There has been a 40% reduction in bed numbers within the past 20 years without any corresponding increased funding for community services. Yet the distribution of those beds remains inadequate for the population needs.

Hospital facilities (Table 1) are mainly old and institutional in nature and located without obvious regard to the location of population centers, with some large and very rural and most isolated from communities and other medical specialty care. The hospital funding arrangements were a counterincentive for psychiatric wards in general hospitals as these produce less income.

**Table 1.** Psychiatric inpatient and outpatient facilities.

Facility	Beds	Day-care places
State Psychiatric Hospitals	2225	128
Mental Health Centers	1032	564
General Hospital Psychiatric Wards	425	48
University Clinics	375	168
NGO	<20	N/A
Military Medical Academy	25	30
Interior Ministry Medical Institute	10	30
Social Service homes for Learning Disability and Dementia	1,036	–

It appears that over 90% of inpatients are voluntarily in hospital, but accurate data were not available. The Panel were unable to develop a clear understanding of whether there were organized patient pathways through the hospital system.

Some outpatient services are run by the State Psychiatric Hospitals, Mental Health Centers and University Psychiatric Clinics. These do not appear to be run according to a national policy or consistent plan, with evidence of great variability in availability, target patient group, form, and service provision.

Most outpatient services are provided by private practice, with most psychiatrists working in both state-funded facilities and in private practice.

### Workforce

The mental health care workforce (Table 2) in Bulgaria is concentrated in urban centers and has been severely depleted by emigration since joining the EU. There are very low numbers of qualified staff, who are disproportionately old, with inadequate current trainee and student numbers, and very poor morale. There was a lack of national strategic workforce planning.

**Table 2.** Mental health workforce in stationary structures 2017 [7].

Psychiatrists	447
Other doctors	71
Mental health nurses	978
Clinical psychologists	89
Social workers	50
Health care assistants	789

The Panel noted the severe shortage of staff, facilities, training, and treatment in Child Psychiatry and Forensic Psychiatry.

Social services are the responsibility of the Ministry of Social Affairs but there was no effective collaboration with the Ministry of Health.

### Financial resources and funding

Funding for inpatient psychiatric care is estimated at about BGN 100 million (equal to EUR 50 millions) or about 2.5% of the total healthcare budget in the country. The latter comprises about 8% of the Bulgarian GDP which is itself below the European average, and in addition, Bulgaria has one of the lowest spends on health per capita in Europe of slightly more than EUR 1,000 per year.^8^

Private outpatient practice is funded by direct payment and limited National Health Insurance Fund reimbursement. The estimated cost of psychopharmacological treatment for psychiatric outpatients eligible for reimbursement is over BGN 35 million a year (equal to EUR 17.5 million). In practical terms reimbursement is covered by National Health Insurance for only four long-acting antipsychotic drugs. On the other hand, outpatient services in the form of consultations are estimated to receive funding of about BGN 5 million a year.

The funding of mental health services in Bulgaria is complex and comes from different streams that do not appear to always be coordinated or in communication, leading to a confused situation with a lack of joined-up care and an incoherent patient pathway. The Panel noted that even senior clinicians were not always in agreement regarding the rules and mechanisms for funding.

Many challenges regarding funding systems in operation were identified, including chronic underfunding, confusion regarding the rules, incentives for longer inpatient stays, and divided responsibilities. Underinvestment in mental health services is causing significant financial harm to the Bulgarian economy due to both the increased costs of treatment and the loss of economic productivity. There appeared to be multiple unintended financial incentives driving the provision of care, for example, poor pay and low levels of reimbursement.

## Challenges and Issues for Bulgarian Mental Health Service Delivery

### Good practice

The Panel were impressed by the open and transparent attitudes that they encountered at all levels from the Deputy Minister through administrators, clinical staff, and patients. We believe that this openness is what will make improvements possible. The Panel were impressed by the passion and commitment of many of the stakeholders whom we met, and in particular their drive to improve services.

The Panel were impressed with the high caliber of psychiatry specialist trainees whom we met and believe that retaining them in Bulgaria will be a key part of future service improvements.

### Poor practice and challenges

The Panel encountered conditions detailed in the main report that appeared to be in breach of European law (Article 3 of the European Convention on Human Rights) and the United Nations Convention on the Rights of Persons with Disabilities.

In addition, the Panel noted that as a result of chronic underfunding there were unacceptable conditions in many of the facilities visited. Examples included very poor built environments in some, lack of adequate staffing, lack of therapeutic activity, overcrowding, lack of national strategic planning, fragmentation of services, lack of quality control and outcome monitoring, and lack of joined-up working including between the Ministries of Health and Social Affairs that was a particular impediment for achieving timely discharge for many patients who required longer term support in the community and/or supported accommodation.

The EPA Panel found that a lack of joined-up planning and accountability was a pervasive problem across the whole mental health care system that had contributed to the impasse with stakeholders locked in disagreements with one another, with high levels of mutual mistrust.

There were significant workforce issues including inadequate numbers of clinical staff, loss of staff due to emigration, and an aging workforce, lack of investment in training, poor morale, and unequal distribution of staff. Salaries were too low, leading to incentives to seek other income sources.

There was longstanding major underinvestment and underfunding in Bulgarian mental health services, especially compared with other medical specialties. Any improvement or meaningful reform would require more investment. However, the economic benefit of reform to Bulgarian society should be considerable with more people off disability benefits, in employment and requiring lower overall healthcare costs. Existing funding mechanisms were not sufficiently coordinated and were complex and confusing. A disproportionate number of people with mental health problems were not covered by the National Health Insurance Fund, which in turn did not pay for many psychiatric investigations and treatments.

Both formal and informal meetings with many stakeholders revealed a substantial lack of consensus between them. This represents a significant impediment to change, and might have contributed to delay, if not avoidance of necessary change. However, the Panel noted that there appeared to be consensus regarding the nature of the challenges faced, and hence the need for change.

The Panel was frequently informed that negative public attitudes had prevented or would prevent change, and in particular were an obstacle to establishing community based mental health services. The EPA Panel wished to challenge this perception. The Global Initiative on Psychiatry had established and runs a successful community-based service and had been able to easily deal with this and had not encountered major difficulty from the local residents or general public. The service is patient-focused, empowering and has successfully tackled and overcome many of the issues that are perceived to be holding back similar service developments elsewhere.

The Bulgarian Government has an “Action plan—National Strategy for Long-Term Care” but the EPA Panel was concerned that there may be insufficient recognition of the financial benefit to the country as a whole of investing in mental health services, and the amount of additional investment required including into preventative services.

Psychiatry and psychiatric services were significantly underfunded and marginalized compared to other medical specialties, often located far away from other medical services and were subject to discrimination and complacency including from the authorities regarding this.

In spite of the attempts to develop strategic programs aimed at stimulating implementation of best practice across Bulgarian mental health services, starting in the 1970s, good practices still seem to stem from either individual efforts or declarative program documents that have been implemented on a limited scale.

## Conclusions and Recommendations

This EPA Panel Report includes 20 recommendations regarding proposed changes and reforms:

1. Appoint a national Clinical Leader with executive operational responsibility and decision-making authority for the change program. The Clinical Leader should be appointed jointly by the Ministries of Health and of Social Affairs and should report directly to the two Ministers.
2. Appoint a national Task Force chaired by this national Clinical Leader to advise, lead and implement the change program.
3. Allocate 10% of the health budget to mental health (needed to urgently address the legacy of serious underfunding).
4. Increase salaries for clinically qualified staff working in mental health care settings; attract trainees and favor the return of those who emigrated to work in more attractive settings.
5. Counteract by appropriate campaigns and initiatives the fear that the reform will translate into further reduction of resources allocated to mental health including inappropriate closure of inpatient beds.
6. Avoid any attempt to import models wholesale from outside; tailor the development of a more community-based mental health system to the specific context of Bulgaria and make full use of local strengths and experience.
7. Implement national action plans to eliminate discrimination and improve attitudes toward people with mental disorders, and improve the image of psychiatrists and the whole mental health workforce.
8. Involve patient and family associations, alongside and together with scientific and professional organizations in planning and implementing the different steps of the reform process.
9. Establish a collaborative and effective working relationship between the Ministry of Health and the Ministry of Social Affairs.
10. Implement training programs for existing staff to enhance skills and improve morale, and support best clinical practice including by older staff. Do not accept poor practice, implement a performance management processes to improve practice and, if needed, replace ineffective staff.
11. Improve education and training in psychotherapy and psychosocial interventions.
12. Improve education and training and significantly increase the number of trainees in all psychiatric specialties including child psychiatry and forensic psychiatry.
13. Plan the implementation and coordination of a realistic spectrum of mental health services responsive to population needs.
14. Develop and implement the action plan for reform of mental health services in such a way that it can be delivered in a step by step manner based on clinical priority and available resources. First in the priority list must be complete reprovision and relocation of services with severe human rights violations. Pilot projects to evaluate viability and gain support are encouraged.
15. Define and monitor strict criteria for involuntary treatment and supported decision making.
16. Provide different but equally humane and high-quality care settings for all patients with mental illness, including old age and child psychiatry, addiction disorders, intellectual disability and forensic psychiatry that are located so as to maximize ease of access for patients and families.
17. Define and require an evidence-based method to measure the quality of services and the outcomes of the reprovision program at the service (e.g., lengths of stay, costs of care, and service quality) and patient level (e.g., recovery, patient satisfaction, and markers of social inclusion). The use of existing standardized quality assessment tools is encouraged (such as the Quality Indicator for Rehabilitative Care which is already translated into Bulgarian and the WHO Quality Rights toolkit).
18. Implement an official and publicly accessible (digital) data platform of relevant and up-to-date quality indicators, compliant with European data protection legislation for surveying, planning, and monitoring the status and progress of the mental health care reforms.
19. Implement an external independent review process to regularly assess progress in implementing change in Bulgarian Mental Health Services.
20. Stimulate and fund research for evidence-based evaluation of implementation, maintenance, adoption, and further development of the reform process.

## Developments since the Report delivery

### Government and political

Since the official publication of the EPA report in 2018 there has been substantial government progress toward implementation:

1. December 2018, the National Assembly (Parliament) of Bulgaria organized a Round Table with stakeholders to deliver the report and possible approaches for delivery of meaningful mental health care reform.
2. This meeting on the EPA report led in March 2019 to the Government appointing a Task Force to produce a new National Strategy for Mental Health and Action Plan for 2020–2030.
3. The Task Force operationalized most of the EPA recommendations, including:
  3.1. Appointment of Joint Standing Committee of Mental Health, affiliated within the Council of Ministers, tasked with coordination and integration of the efforts from the various organizations involved, most prominently the Ministry of Health and the Ministry of Social Affairs.
  3.2. Decision-making responsibility will be granted to this Committee, which will advise regarding allocation of resources both from National Budget and from Operational Programs of the European Commission (already negotiated for the upcoming EU framework budget) in order to support effective mental health reform in Bulgaria.
  3.3. Establishment of centers for community-based based on regional needs assessments with transformation of the hospital services into smaller acute care units to cover particular populations.
  3.4. Introduction of early intervention services.
  3.5. Introduction of quality control measures.
  3.6. Improvement in education and training of mental health personnel.
  3.7. Prioritize increasing salaries.

The Strategy was due by the end of 2019 and will be incorporated into the global National Health Strategy.

The Government has decided to allocate additional funds from the 2019 budget for significant improvement of existing hospital settings considered to be in critical condition.

The Advisory Board for Psychiatry, operational under the Office of the Minister of Health, has produced a new Standard for Psychiatry, currently under revision, which incorporates many instruments and guidelines to further implement the EPA recommendations.

### Organizational

In May 2019, the two main psychiatric organizations in Bulgaria (Bulgarian Psychiatric Association and the College of Private Psychiatry) in collaboration with the Bulgarian Family Carer Association of People with Mental Illness (BGFAMI) issued a joint statement in support of the main recommendations of the report stating:

*"On this basis we propose that the Ministry of Health forms a working group, constituting of experts from the two professional organisations as well as a representative of the Bulgarian patient organisation (Bulgarian Family Carer Association of People with Mental Illness, BGFAMI), which is to be tasked in a period of three months to prepare a plan for action based on the recommendations by EPA….*

*We propose that the working group constitutes of seven experts (three from each professional organisation and one representative of the patient organisation) and that their goal is the generation of a comprehensive vision of the psychiatric reform in Bulgaria. The decisions of the working group will be made on a principle of unanimousness."*

*In June 2019 the College of Private Psychiatry has been duly approached for consultation and coordination of joint effort by the Government Task Force on National Strategy for Mental Health Care 2020–2030 as mentioned above.*

### Final remarks

The mission of the European Psychiatric Association in Bulgaria in 2018 came in after two decades of organizational crisis in psychiatric services in the country. This situation has been further complicated by disagreement among key stakeholders, including nongovernmental professional organizations (NGOs), as to what measures would be appropriate to shape a meaningful reform in mental health care.

The EPA mission revealed a number of concerns in the system and addressed most of them in terms of report recommendations. The Government responded to the EPA report at the highest political level of decision-making by charging a newly appointed Task Force with the task of designing a National Strategy and Action Plan for Mental Health Care.

The EPA mission succeeded in stimulating a productive dialogue between decision making bodies, NGOs, and service users. The main ingredients for this success were clear questions and answers, careful listening to the different opinions, attempts to moderate conflicts and turn suspiciousness and consequent conservative attitudes into constructive proposals for actions that could no longer be postponed.

The process described in the EPA position paper clearly demonstrates, once again, that stakeholders concerned with mental health need to work together and “*speak with one voice*” in order to effect a significant change in the system of psychiatric care delivery.

## Data Availability

The data that support the findings of this study are available from the lead author Julian Beezhold.
